# Gene therapy for presenilin mutation carriers

**DOI:** 10.1002/alz70859_098883

**Published:** 2025-12-25

**Authors:** Inmaculada Sanjuan Ruiz

**Affiliations:** ^1^ VIB Ctr for brain and disease and KULeuven, Leuven, brabant Belgium

## Abstract

**Background:**

Young‐onset dementia, although rare, has a profound impact on patients and their families. One significant form, Autosomal Dominant Alzheimer’s Disease (ADAD) is caused by dominantly inherited mutations in the PSEN1, PSEN2, or APP genes. Mutations of the presenilin gene cause a shift to longer Aβ peptides which aggregate faster. Importantly, there is a linear correlation between age of onset of dementia and how the mutations affect the short over long ratio of Aβ peptides. Current therapies offer only symptomatic relief and fail to address the genetic basis of the disease. Our goal is to develop a highly precise and safe genetic therapy for these patients that addresses the root cause.

**Method:**

We have obtained iPSCs from heterozygous PSEN1 A431E carriers and developed a novel Psen1 A431E mouse model that develops Aβ plaques. We aim to design highly selective antisense oligonucleotides (ASOs) targeting a single nucleotide change in the allele carrying the PSEN1 A431E mutation, while sparing the normal PSEN allele's function. Preventing disease progression will be validated in vivo in our mouse models.

**Result:**

Neurons derived from two PSEN1 A431E lines were used to screen several ASOs targeting the mutation. Mutant mRNA levels were significantly knocked‐down, while sparing the WT allele expression, showing high selectivity with a single nucleotide difference can be reached. Two WT isogenic lines were generated as controls to assess the therapeutic effect of the treatment on the short over long Ab ratio. Upon ASO treatment, a significant decrease of long Aβ42 and Aβ43 species along with a significant increase of short Aβ37 and Aβ38 species resulted in a shift in Aβ ratio towards WT control Aβ profile. In vivo treatment with Psen1 A431E targeting ASOs in mice similarly showed high selectivity and significant target knock‐down and shift in the Aβ profile by decreasing long Aβ species.

**Conclusion:**

We provide proof of concept that it is possible to generate active ASOs selectively targeting mutant PSEN1, addressing the genetic root cause of ADAD. Such gene therapy approach could be applied to all ADAD causing mutations and potentially addressing the disease before symptoms appear, delaying or even preventing the progression of pathology.

